# What happens post‐lockdown for people with disability? Autonomy, quality of life, service access and health changes for people with spinal cord injury in Victoria, Australia after COVID‐19 social distancing restrictions

**DOI:** 10.1111/hsc.13958

**Published:** 2022-08-04

**Authors:** Ali Lakhani, Salvatore Dema, Josh Hose, Nazim Erdem, Dennis Wollersheim, Peter Grimbeek, Susan Charlifue

**Affiliations:** ^1^ The School of Psychology and Public Health La Trobe University Melbourne Victoria Australia; ^2^ The Hopkins Centre, Menzies Health Institute Queensland Griffith University Meadowbrook Queensland Australia; ^3^ Palliative Care Department Eastern Health Wantirna Victoria Australia; ^4^ Austin Health ‐ Royal Talbot Rehabilitation Centre Kew Victoria Australia; ^5^ AQA Victoria Heidelberg Victoria Australia; ^6^ Upper Brookfield Brisbane Queensland Australia; ^7^ Craig Hospital Englewood Colorado USA

**Keywords:** autonomy and participation, COVID‐19, disability, health service access, longitudinal, social distancing

## Abstract

Social distancing restrictions are undoubtedly important for controlling the spread of COVID‐19 however, they are also adversely impacting population health and health service access. It is important that priority populations with a disability which may already have adverse health, access to health services, and autonomy and participation compared to those without disability, are able to receive preventative health and social care during periods of restriction. The impact of social distancing restrictions on people with disability is not uniform nor well‐understood. Research has been cross‐sectional and considered data gathered during social distancing restrictions, or longitudinal, considering data gathered during a pre‐pandemic baseline. This longitudinal study investigated the impact of lifting social distancing restrictions on priority domains for people with disability including autonomy and participation, access to health services, health issues and quality of life. People with spinal cord injury in Victoria, Australia (*n* = 71) completed a survey towards the end of social‐distancing restrictions (T1) and 6‐months post social distancing restrictions (T2). Non‐parametric tests for significant differences confirmed that 6‐months post‐lifting social distancing restrictions participants experienced a significant increase in health conditions, a significant decrease in the number of inaccessible health services, and a significantly lower level of limitations across participation and autonomy, outdoor autonomy and work and education domains. QOL improved 6‐months post lifting restrictions, however not to a significant level. The adverse health experienced by people with spinal cord injury after lifting restrictions may in part result from limited health service access and reduced participation during the time of restrictions. Clear definitions of what constitutes as essential care may ensure that eligible and required care remains received during lockdown or instances when service provision is compromised. Health and social care providers should be equipped with the knowledge of priority populations so that their support can be targeted to those most in need.

**What we know**

- Social distancing restrictions control the spread of COVID‐19 however, can also adversely impact population health.
- Compared to a period prior, health service access and quality of life are poorer during the pandemic.
- The impact of lifting social distancing on quality of life, health service access and health issues remains unclear.

**What the paper adds**

- Compared to a period where social distancing restrictions have been lifted, health service access for people with spinal cord injury during social distancing is poorer.
- Secondary conditions increase after lifting restrictions and this is potentially due to limited health service access and reduced participation and autonomy during social distancing.
- Health and social care providers need to be resourced to provide targeted care for people with disability under the context of COVID‐19 social distancing restrictions.

## 1 INTRODUCTION

The COVID‐19 pandemic has had an impact on global health. Countries have undertaken diverse measures to reduce the spread of COVID‐19, including social distancing restrictions and stay‐at‐home orders (Tuijt et al., 2021). Various groups are impacted by the pandemic and the consequences for all groups are not uniform nor well‐understood. For example, people living with a chronic disease report higher levels of fear of contracting COVID‐19 (Korukcu et al., 2021), parent stress has increased during the pandemic (Freisthler et al., 2021), and for young people, mood, stress and substance use have been adversely impacted as a result of social distancing restrictions (Emery et al., 2021).

A combination of factors (e.g. increased COVID‐19 cases and loss of employment) are contributing to adverse health outcomes during the pandemic, and social distancing restrictions also play a critical role (Devaraj & Patel, 2021). Knowledge gaps surrounding the impact of social distancing restrictions on health status and health service use exist. For example, our understanding of the long‐term impact of the pandemic (Sachser et al., 2021), particularly after the lockdown has ceased, is unclear. Additionally, limited research has focused on the impact of social distancing restrictions on the health and well‐being of people with disability (Okonkwo et al., 2021).

Preliminary research confirms that social distancing restrictions may protect people with disability from infection, whilst simultaneously worsening conditions for some (Courtenay & Perera, 2020). People with intellectual and developmental disability have experienced worsened quality of life outcomes during the pandemic (Friedman, 2021). Compared to people without disability, people with disability have had significantly higher stress levels and greater difficulty accessing health services during the pandemic (Okoro et al., 2021). Problematically, this inability to access health services may be contributing to long‐term adverse physical and mental health outcomes (Theis et al., 2021). It is essential that research continues to investigate the impact of the COVID‐19 pandemic, and consequences of the pandemic, on the health and well‐being of people with disability. Such research needs to consider the needs of groups with distinct conditions (Turk & McDermott, 2020), and a disability‐inclusive COVID‐19 response is required (Kuper et al., 2020).

There is an emerging body of literature investigating the impact of COVID‐19 and social distancing restrictions on the health and well‐being of people with spinal cord injury (SCI). The most robust research is longitudinal and has compared health and well‐being outcomes during the pandemic to a pre‐pandemic baseline. Findings are somewhat mixed however generally find adverse health and well‐being during the pandemic compared to prior. Elaraby et al. (2022) found that compared to pre‐pandemic levels, during the pandemic, people with SCI report significantly lower physical and psychological health, and social relationships. Whilst García‐Rudolph et al. (2021) found that people with SCI reported significantly lower levels of social integration and significantly higher levels of depression during the pandemic compared to prior. They also found that a younger group of people with SCI reported significantly lower physical and psychological health whilst an older group of people did not report significantly lower levels across these domains. Cross‐sectional work has confirmed that during the pandemic people with SCI experience poorer access to healthcare (Hearn et al., 2021; Vives Alvarado et al., 2021), and increased secondary health conditions (Hearn et al., 2021).

Targeted support to address the health and well‐being of people with disability post‐COVID‐19 is necessary. Informing targeted support efforts requires a better understanding of the impact of the pandemic on priority domains for people with disability and particularly people with SCI including: autonomy and participation (Marco‐Ahulló et al., 2021), access to health services (Hearn et al., 2021; Vives Alvarado et al., 2021), overall health issues (Hearn et al., 2021) and quality of life (Elaraby et al., 2022).

Most research that has investigated the impact of the pandemic on people with disability and particularly people with SCI has been cross‐sectional and assessed data gathered during social distancing restrictions and a heightened number of cases, or longitudinal, using data gathered prior to the pandemic (Bignardi et al., 2020; Kim et al., 2020; Magson et al., 2021; Pierce et al., 2020). Such research has been incredibly useful and confirmed that the pandemic has had an impact on the health and well‐being of people with and without disability. However, the long‐term consequences of the pandemic on the health and well‐being of people with disability are unclear. Research needs to move beyond longitudinal designs where data gathered during the pandemic is compared to retrospective data, and instead compare data gathered during the pandemic and social distancing restrictions with data collected after restrictions have ceased. Such research will confirm which domains will recover or worsen after the pandemic and inform the delivery of responsive health and social care efforts.

### 1.1 The current study

The current longitudinal study aimed to investigate the impact of lifting social distancing restrictions on priority domains for people with SCI residing in the state of Victoria, Australia. The research question, priority domains considered, and methodology was informed by the literature and the investigator team which included peer‐support workers with lived experience of SCI from an organisation that provides health and social support to people with SCI. The study was designed to answer the following question:

1. What is the impact of lifting social distancing restrictions on the availability of health services, health conditions, quality of life, and participation and autonomy of people with SCI living in Victoria, Australia?

The study was informed by the following hypotheses:

H1: As quality of life outcomes are poorer during COVID‐19 restrictions (Friedman, 2021), quality of life improves subsequent to lifting social distancing restrictions.

H2: As access to health services is adversely impacted during COVID‐19 restrictions (Connor et al., 2020; Dalise et al., 2021; Okonkwo et al., 2021; Okoro et al., 2021), there will be fewer services identified as inaccessible after lifting social distancing restrictions.

H3: As adverse health outcomes are theorised to emerge as a result of poorer access to health services during COVID‐19 restrictions (Theis et al., 2021), there will be increased adverse health outcomes which only become identifiable subsequent to lifting social distancing restrictions.

H4: As COVID‐19 restrictions have an adverse impact on participation and autonomy (Ammar et al., 2020), autonomy and participation will improve subsequent to lifting social distancing restrictions.

Australian‐centric COVID‐19 research provides the unique opportunity to develop an understanding of the impact of the pandemic for people with disability post‐social distancing and lockdown restrictions. Australia has had a lower COVID‐19 prevalence compared to other western countries (Johns Hopkins Coronavirus Resource Centre, 2020). In June 2020, most states and territories lifted social distancing restrictions which were initiated in March 2020 (Storen & Corrigan, 2020). The state of Victoria was the only exception, where social distancing restrictions were briefly lifted in June 2020, only to be re‐instated from the beginning of July 2020 (Premier of Victoria, 2020b) until the end of October 2020 (Department of Health and Human Services, 2020b) as a result of increased cases. The extent of cases necessitated the ‘state‐of‐disaster’ declaration on 2nd August 2020 (Department of Health and Human Services, 2020a), and the implementation of Stage 4 restrictions for metropolitan Melbourne (Department of Health and Human Services, 2020a). Under these restrictions, residents could leave home only for four essential reasons including: essential shopping, receiving or providing care, work or exercise (up to 1‐h per day). All exercise and shopping were to be conducted within a 5‐km radius of a resident's home. Furthermore, no visitors were permitted at home (unless for essential reasons) and a curfew was instated (see Victoria State Government, 2020b for Stage 4 restriction details). For most of the stated period—from the beginning of August (Department of Health and Human Services, 2020a) to mid‐September (Premier of Victoria, 2020a) ‐ residents within regional Victoria lived under Stage 3 restrictions, which were comparable to Stage 4 restrictions. Stage 3 restrictions did not include a curfew nor a travel radius restriction (see [Victoria State Government, 2020a] for details), however, a resident could only leave home for the four essential reasons. From October 2020 to April 2021, Victoria reported a limited number of COVID‐19 cases (Australian Government Department of Health, 2021) and lived without Stage 3 and Stage 4 restrictions for an extended period.

## 2 METHODS

The La Trobe University Human Research Ethics Committee provided approval to conduct this research (protocol ID: HEC20197).

### 2.1 Study design

A longitudinal design was employed, and participants completed an online survey at two time points. Baseline data (T1) were collected during September and October 2020. During this time, participants were requested to respond to questions given their experience since the commencement of COVID‐19 restrictions within the state of Victoria in March 2020. For most of the referenced six months, participants were experiencing some form of social distancing restrictions (with participants experiencing Stage 4 and Stage 3 restrictions during the latter part of the 6 months). Follow‐up data were collected between April 2021 and May 2021 (T2) and participants were requested to respond to questions given their experience over the previous 6‐months. This 6‐month period coincided with a time where social distancing restrictions had been lifted (Stage 3 and Stage 4 social distancing restrictions gradually ended and with an opening up of commercial, public and health services during September and October 2020). As the current study aimed to investigate the impact of lifting social distancing restrictions on the availability of health services, health conditions, quality of life, and participation and autonomy of people with SCI living in Victoria, Australia, the data collection periods are considered suitable. Specifically, comparing data collecting during (i) a period of increased restrictions (T1) to (ii) a subsequent period with lifted restrictions (T2) allows for the impact of lifting restrictions to be established.

### 2.2 Participants and recruitment

Members and clients from a health and social service organisation providing targeted support to people with SCI were recruited to participate in this study. The organisation provides peer support, advocacy support, daily living and personal care and skill development and capacity‐building support. Participants were recruited via a personalised email (*n* = 1100). One hundred and twenty‐seven people completed the T1 survey and 71 completed the T2 survey. Demographic information has been included in Table 1.

**TABLE 1:** Demographic information

Domain	Frequency/mean (SD)
Sex	
Male	52
Female	18
Age (years)	55.81 (12.25)
Education	
Tertiary	49
Highschool or under	21
Employment status	
Employed or engaged in activity (working full‐time or part‐time, or volunteering)	38
Retired	17
Unemployed	15
Home ownership status	
Home Owner	56
Renting or social housing	12
Household composition	
Family and/or friends	54
Alone	16
Primary health condition	
Paraplegia	35
Tetraplegia	31
Years with condition	16.30 (14.07)

### 2.3 Measures

Questions and measures were identified via a collaborative approach to identify research priorities amongst university researchers and people with lived experience of disability (Lakhani, et al. 2021). Outcome data included measures for participation and autonomy, quality of life, health service access, and health conditions. In relation to autonomy and participation, participants were requested to complete the Impact on Participation and Autonomy Questionnaire [IPAQ] (Cardol, De Jong, & Ward, 2002). The IPAQ measures the extent of difficulties that people with chronic conditions and/or neurological disability have across five domains: Autonomy Indoors, Autonomy Outdoors, Family Role, Social Life and Relationships, and Work and Education. A higher score is indicative of having greater difficulties. The IPAQ has been used amongst people with SCI (Bombardier et al., 2016; Cardol et al., 2001; Cardol, de Jong, van den Bos, et al., 2002; Craig et al., 2015). Cronbach's alphas were calculated for T1 values and comparable to Cardol et al. (2001), they produced values indicative of subscales being reliable (see alpha in brackets): Autonomy Indoors (0.91), Family Role (0.89), Autonomy Outdoors (0.84), Social Life and Relationships (0.86), Work and Education (0.90). Quality of life was measured using the International Spinal Cord Injury Quality of Life (QOL) measure (Charlifue et al., 2012)—a reliable measure of QOL for people with SCI (New et al., 2019). The measure consists of three questions which measure satisfaction across three domains: physical health, psychological health, and overall well‐being. Comparable to findings by New et al. (2019), Cronbach's alpha calculated for the T1 value confirmed that the scale is reliable (0.83).

Health service access was measured by summing the number of health services participants indicated being unable to access over the reference period. These services included: general practitioners, urologists, occupational therapists, physiotherapists, specialised rehabilitation, masseur, osteopath and podiatrist. Additionally, participants were able to indicate whether they were able to access two local SCI services. The health and social service organisation recruiting participants did not provide these services and was not listed as one of the local specialist SCI services. A higher number was indicative of being able to access fewer services. Similarly, health issues were measured by summing the number of health issues a participant indicated having over the reference period. These issues were informed by Kalpakjian et al. (2007) and included: neuropathic pain, sexual dysfunction, joint contractures, spasticity, urinary tract infection, shoulder problems, bowel incontinence, weight problems, urinary incontinence, trouble sleeping, elbow/wrist problems, neurological deterioration, fatigue, pressure ulcers, constipation, injuries caused by a loss of sensation, lightheadedness/dizziness, respiratory infections, autonomic dysreflexia, thrombosis/embolism and/or kidney/bladder stones.

### 2.4 Data analysis

IBM SPSS 25 was used for all analyses. Shapiro–Wilk tests were performed for T1 and T2 outcome variables to establish whether they were normally distributed and whether parametric or non‐parametric tests should be used. As clarified in Table 2, with the exception of Autonomy Outdoors, *p*‐values were indicative of a non‐normal distribution for each outcome during at least one time period. Thus, non‐parametric tests were used for all analyses (as the T2 *p*‐value for Autonomy Outdoors trended towards significance, it was deemed appropriate to also implement non‐parametric tests for this variable).

**TABLE 2:** Shapiro–Wilk *p*‐values for outcome variables

Item	*p*‐value	
	T1	T2
Quality of life	<0.05	<0.05
Impact on participation and autonomy	0.066	<0.05
Autonomy indoors	<0.001	<0.001
Autonomy outdoors	0.146	0.054
Family role	<0.05	<0.05
Social life and relationships	0.081	<0.05
Work and education	0.052	<0.05
Health issues	<0.05	0.204
Health service access	<0.001	<0.001

Forty‐one participants were located in metropolitan Victoria (subject to Stage 4 restrictions), 26 participants were located in regional Victoria (subject to Stage 3 restrictions) and 4 participants did not provide an address. The Mann–Whitney test statistic was produced to establish if there were significant differences in T1 outcome values between participants residing in metropolitan Victoria, and regional Victoria. Differences were non‐significant across all outcomes with exception of Work and Education (U = 189.50, *p* < 0.05). As a result, subsequent analyses involved the entire sample.

Wilcoxon signed‐rank tests were used to test for a difference in quality of life, participation and autonomy, health issues, and health service use between both time periods. Where differences in health issues and health service use were apparent, McNemar's test statistic was used to test for significant differences for each health issue, and each health service,between the two time periods.

### 2.5 Findings

**FIGURE 1:**
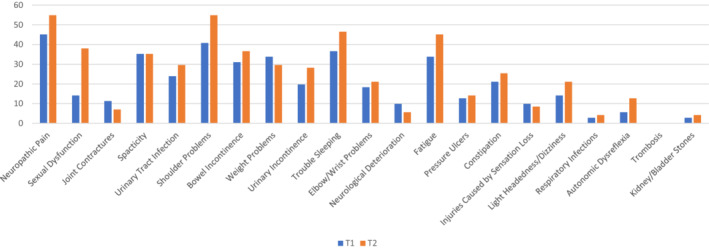
Percentage of participants experiencing secondary health condition.

**FIGURE 2:**
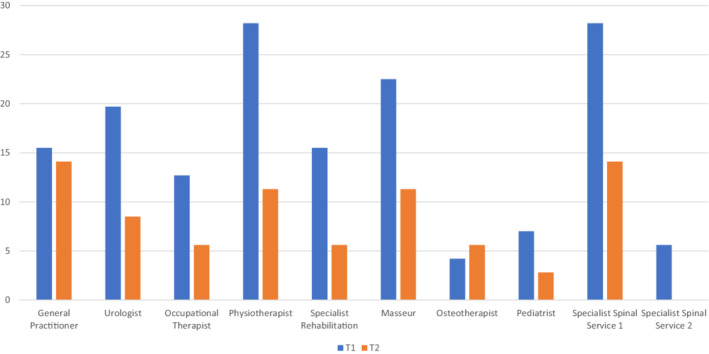
Percentage of participants indicating a health service was inaccessible.

Table 3 includes descriptive statistics for all outcome variables. In relation to participation and autonomy measures, with the exception of Family Role, mean and median values were higher during T1, indicative of participants having greater limitations across participation and autonomy domains whilst in lockdown. In relation to QOL, mean and median values were higher during T2, indicative of participants having an improved QOL once exiting lockdown. In relation to health issues and health service access, mean and median values suggest that participants had greater health issues whilst improved access to health services during T2. Figure 1 illustrates the percentage of participants indicating each health issue they had during T1 and T2. Generally, a greater percentage of participants indicated having each health issue during T2. Figure 2 illustrates the percentage of participants indicating health services which were inaccessible during T1 and T2. Generally, fewer participants indicated inaccessible health services during T2.

**TABLE 3:** Descriptive statistics for all outcome variables

Time 1								Time 2						
Domain	Min.	25th percentile	Median	75th percentile	Max.	Mean	SD	Min.	25th percentile	Median	75th percentile	Max.	Mean	SD
IPAQ	32	56	68	81	137	69.28	18.99	36	50.5	58	74.5	125	63.66	19.96
Autonomy indoors	7	7	9	13	31	10.5	4.2	7	7	10	15	29	11.3	4.9
Family role	7	10	14	19	34	14.8	5.7	7	10	14	18	27	14.6	5.3
Autonomy outdoors	5	11	14	19	25	14.9	4.6	5	9	11	14	25	11.6	4.4
Social life and relationships	7	10	14	17	24	14.0	4.6	7	9	13	17	25	13.4	4.9
Work and education	6	12	15	21	31	15.9	6.2	6	11	13	16	30	14.1	5.7
QOL	0	16	21	23	30	19.4	5.4	3	16	22	24	30	20.1	5.8
Health issues	0	1	4	5	11	3.6	2.6	0	3	5	7	13	5.2	2.6
Health service access	0	1	2	3	9	1.9	1.7	0	0	0	1	6	0.8	1.3

Table 4 includes Z scores and P‐values from Wilcoxon signed‐rank tests. There were significant differences between T2 and T1 values for the following: Impact on Participation and Autonomy, Autonomy Outdoors, Work and Education, Health Issues, and Health Service Access. Median values indicate that during the six months post lockdown (T2) participants had significantly (i) lower Participation and Autonomy limitations (ii) lower Autonomy Outdoors and Work and Education limitations, (iii) a greater number of health issues and (iv) fewer health services which were inaccessible. The effect of living six months post lockdown, given Cohen's classification, was large for autonomy outdoors (0.66), health issues (0.53), health service access (0.57), and moderate for work and education (0.45) and participation and autonomy as a whole (0.39). Significant differences between T1 and T2 outcome values for autonomy indoors, family role, social life and relationships and quality of life were not apparent.

**TABLE 4:** Z‐scores and *p*‐values from Wilcoxon signed‐rank tests

Outcome	Z‐score	*p*‐value
Impact on participation and autonomy	−2.481	*p* < 0.05
Autonomy outdoors	−5.392	*p* < 0.001
Autonomy indoors	−0.889	*p* = 0.374
Family role	−0.189	*p* = 0.850
Social life and relationships	−1.755	*p* = 0.079
Work and education	−3.136	*p* < 0.05
Health issues	−4.471	*p* < 0.001
Health service access	4.800	*p* < 0.001
Quality of life	−1.339	*p* = 0.181

McNemar's test statistic was used to test for significant differences between T1 and T2 values for each health issue and health service. Tables 5 includes the number of people with a particular health issue during and after social distancing restrictions, whilst Table 6 includes the number of people who found health services accessible and inaccessible during and after social distancing restrictions. As clarified in Table 5, the proportions of people experiencing sexual dysfunction, and shoulder problems between T1 and T2 were significantly different. A greater proportion experienced both health issues during T2. As clarified in Table 6, the proportions of people indicating that urologist, physiotherapist and spinal health services were available between T1 and T2 were significantly different. A greater proportion of participants indicated that the suggested services were available during T2.

**TABLE 5:** Cross‐tabulation table clarifying secondary health issues during both periods

During social distancing restrictions	After social distancing restrictions		*p*‐value
	Health issue not present	Health issue present	
Neuropathic pain			0.092
Health issue not present	29	10	
Health issue present	3	29	
Sexual dysfunction			0.002
Health issue not present	39	22	
Health issue present	5	5	
Joint contractures			0.549
Health issue not present	59	4	
Health issue present	7	1	
Spasticity			1
Health issue not present	37	9	
Health issue present	9	16	
Urinary tract infection			0.481
Health issue not present	43	11	
Health issue present	7	10	
Shoulder problems			0.031
Health issue not present	28	14	
Health issue present	4	25	
Bowel incontinence			0.541
Health issue not present	35	14	
Health issue present	10	12	
Weight problems			0.664
Health issue not present	38	9	
Health issue present	12	12	
Urinary incontinence			0.146
Health issue not present	48	9	
Health issue present	3	11	
Trouble sleeping			0.21
Health issue not present	30	15	
Health issue present	8	18	
Elbow/wrist problems			0.815
Health issue not present	48	10	
Health issue present	8	5	
Neurological deterioration			0.375
Health issue not present	63	1	
Health issue present	4	3	
Fatigue			0.169
Health issue not present	30	17	
Health issue present	9	15	
Pressure ulcers			1
Health issue not present	56	6	
Health issue present	5	4	
Constipation			0.607
Health issue not present	47	9	
Health issue present	6	9	
Injuries caused by loss of sensation			1
Health issue not present	59	5	
Health issue present	6	1	
Light headedness/dizziness			0.302
Health issue not present	51	10	
Health issue present	5	5	
Respiratory infections			1
Health issue not present	66	3	
Health issue present	2	0	
Autonomic dysreflexia			0.125
Health issue not present	61	6	
Health issue present	1	3	
Thrombosis/embolism			NA
Health issue not present	71		
Health issue present			
Kidney/bladder stones			1
Health issue not present	66	3	
Health issue present	2	0	

**TABLE 6:** Cross‐tabulation table clarifying inaccessible health services during both periods

During social distancing restrictions	After social distancing restrictions		*p*‐value
	Accessible	Inaccessible	
General practitioners			1
Accessible	54	6	
Inaccessible	7	4	
Urologist			0.039
Accessible	55	2	
Inaccessible	10	4	
Occupational therapist			0.227
Accessible	59	3	
Inaccessible	8	1	
Physiotherapist			0.008
Accessible	48	3	
Inaccessible	15	5	
Specialised rehabilitation			0.065
Accessible	58	2	
Inaccessible	9	2	
Masseur			0.096
Accessible	50	5	
Inaccessible	13	3	
Osteotherapist			1
Accessible	66	2	
Inaccessible	1	2	
Podiatrist			0.453
Accessible	64	2	
Inaccessible	5	0	
Spinal health service (1st local)			0.031
Accessible	47	4	
Inaccessible	14	6	
Spinal health service (2nd local)			NA
Accessible	67		
Inaccessible	4		

## 3 DISCUSSION

This study aimed to investigate the impact of lifting social distancing restrictions on priority domains for people with SCI residing in the state of Victoria. Access to health services was adversely impacted during COVID‐19 restrictions and the hypothesis (informed by Connor et al., 2020; Dalise et al., 2021; Okonkwo et al., 2021; Okoro et al., 2021) was upheld. In this regard, a significantly greater proportion of participants indicated that physiotherapists and urologists were not accessible during lockdown restrictions. These health services often require face‐to‐face visits, and/or their practices benefit from information gathered from scans requiring in‐person attendance (i.e. X‐rays or MRI), thus the ability to access these services may have been compromised. The finding that people with SCI experience limited access to health services during a period of lockdown is not surprising and has been confirmed elsewhere (see Gustafson et al., 2021). People with SCI often have secondary conditions that need to be addressed, and their ability to access such services safely, during the time of restrictions resulting from a pandemic, should be prioritised. As restrictions still allowed people to receive essential medical care, it could be that those with SCI were eligible to receive these services in person, but they decided otherwise and/or the provider advised against it. Care for populations who are at risk of developing secondary conditions should be considered essential. Educating health service providers and end users around their eligibility to access services during the time of lockdown need to be prioritised. Health departments providing clear definitions of essential care can assist to avoid instances where eligible and required care goes unreceived during a lockdown or other instances where service provision is compromised.

Health issues significantly increased subsequent to lockdown restrictions and the hypothesis informed by Theis et al., 2021 was upheld. Health issues may have emerged as a result of poor access to health services during the period of lockdown. A significantly greater proportion of participants indicated having shoulder problems and sexual dysfunction subsequent to lockdown. These health issues correspond with health services which were identified as inaccessible; specifically physiotherapists and urologists. These results should be treated with caution as the referenced health conditions may have emerged post‐lockdown due to another reason (i.e. increased physical activity post‐lockdown may have contributed to shoulder problems). Regardless, as research has confirmed that poor access to health services during the pandemic can contribute to increased secondary health issues for people with SCI, it is possible that the referenced conditions were a result of being unable to receive allied health and specialist support. For example, a survey of rehabilitation clinicians confirmed that during the pandemic clients with SCI had limited access to essential health services, and experienced increased medical complications (Gustafson et al., 2021). Furthermore, cross‐sectional work eliciting the opinions of people with SCI during the pandemic has confirmed poor access to health services and increased secondary complications as concerns (Hearn et al., 2021). In their study of health‐related QOL amongst people with SCI prior to, and during the pandemic, Matsuoka and Sumida (2021) found that people with SCI reporting lower health‐related QOL had fewer home nursing and rehabilitation service visits compared to a group where health‐related QOL remained unchanged.

In response, it is important that health and social services are prepared to address the potential increase in health issues, specifically health issues for priority populations which have a greater likelihood of developing secondary health issues, subsequent to lockdown and social distancing restrictions being lifted. In this respect, providers should be resourced to handle increased cases, and ensure that they have the knowledge base to address complex secondary issues. Furthermore, the potential psychological consequences of experiencing these secondary issues should not be overlooked, and an integrated public health approach to addressing secondary issues needs to be employed. In this respect, allied health providers and specialist providers should work in concert to ensure that those who had health issues potentially resulting from inaccessible health services during the pandemic are able to receive adequate care.

People with neurological disability are more likely to have secondary health conditions (Foster et al., 2015). The fact that social distancing restrictions can contribute to these conditions suggests that healthcare systems need to develop better practices to support people with disability or people with a greater risk of developing secondary conditions. This is especially the case during the time of a pandemic. It is argued that telehealth may be a worthwhile method to address the health consequences of the pandemic for people with SCI (Elaraby et al., 2022; Hearn et al., 2021). Telehealth has certainly been a valuable health service delivery method during the period, evidenced by an increase in telehealth delivery amongst rehabilitation physicians who work with people with traumatic injury, including SCI (Gustafson et al., 2021). Despite the value of telehealth, a recent survey of rehabilitation physicians confirmed that over half felt as though the delivery method did not meet their patients' distinct needs (Gustafson et al., 2021). Consequently, ensuring health service access remains an important goal moving forward (Gustafson et al., 2021), and such access should allow for a combination of delivery options to ensure that patient needs are met.

The findings supported the hypothesis that lockdown restrictions have an adverse impact on autonomy and participation amongst study participants. In this respect, participants experienced significantly greater limitations in outdoor autonomy and work and education domains during the lockdown. Outdoor autonomy within the IPAQ is measured with five questions which generally consider (i) meeting people outside of the home and (ii) enjoying leisure the way desired. Greater limitations within this domain during the period of lockdown have been confirmed by cross‐sectional research which found that experiencing social distancing restrictions adversely impacts social participation (Ammar et al., 2020). Developing an understanding around the adverse consequences resulting from social participation limitations can inform health and social service providers to best support the needs of people during and subsequent to lockdown. For example, if the lack of social participation contributes to adverse mental health outcomes (Kim et al., 2021), perhaps mental health consultations during lockdown would be a proactive and preventative health service approach. It is important that priority populations with certain conditions which may already have adverse autonomy and participation compared to those without are prioritised to receive preventative health and social care. Health and social care providers should be equipped with the knowledge of priority populations so that their support can be targeted to those most in need. In this respect, once again, the health departments have a role to play, as their ability to convey this information throughout diverse practitioners across health systems can ensure that providers are aware of groups which may require the most support.

In relation to employment and work, the IPAQ includes six questions which generally measure the ability of someone to obtain and keep work. The current study findings indicating that experiencing a lockdown can adversely impact employment also has been confirmed (Bauer & Weber, 2021). People with disability already face adverse employment consequences (Mizunoya & Mitra, 2013), so it is important that subsequent to the pandemic, employment service providers provide targeted support for these individuals. This can be in addition to, or in conjunction with, strategies utilised by health and social care providers broadly to support employment outcomes of people with disability.

Significant differences in autonomy and participation domains including family role, social life and relationships, and autonomy indoors were not apparent. In some respects, this appears to be reasonable given the nature of the measures used for these domains. Within the IPAQ, Family Role measures the ability to complete tasks within the home and Autonomy Indoors measures self‐care domains (i.e. dressing). As such, lockdown restrictions may not have impacted the ability of participants in these areas . Within the IPAQ, Social Life and Relationships generally measure the quality of relationships and respect received. Significant differences across this domain may not have been apparent as the location (i.e. outdoor location) was not considered and the quality of relationships may not have been impacted.

The hypothesis that subsequent to lifting COVID‐19 restrictions, QOL would significantly improve was not upheld. QOL did improve, but not to a significant level. It is possible that QOL outcomes amongst the sample were not considerably impacted by social distancing restrictions. Alternatively, there is the potential that QOL outcomes would make further improvements over time but these were not apparent during the 6‐month follow‐up. Previous research by Elaraby et al. (2022) confirmed that QOL during the pandemic was significantly lower for people with SCI compared to a prior period. Taken together, findings suggest QOL is better prior to and subsequent to, social distancing restrictions. The findings confirm that social distancing restrictions certainly adversely impact QOL, however also suggest that the impact of social distancing restrictions on QOL may be short‐lived and reversible. Long‐term studies are needed to confirm the long‐term impact of social distancing restrictions on people with disability. Furthermore, it is important that further studies investigate if access to health and social care, and health issues, are associated with these QOL outcomes.

This study has limitations which are important to consider. This study investigated the impact of lifting social distancing restrictions on the health and well‐being of people with SCI in the state of Victoria. At the time of data collection, the state of Victoria experienced some of the most robust social distancing restrictions globally. As this is the case, the extent of health and health service use consequences may be specific to places where similar public policy measures have been employed. The study included a population of people with SCI, and thus the findings may be generalisable to other groups who have experienced a traumatic injury, people with neurological disability, and/or ageing adults who use a mobility aid. Finally, the findings from this research included a population of 71 and produced significant findings. Replicating the study with a larger sample is warranted.

## 4 CONCLUSION

Social distancing restrictions have an adverse impact on health service use, autonomy and participation, and health outcomes of people with SCI. Access to health and social services subsequent to the removal of social distancing restrictions may remedy some of these adverse consequences. It is important that future research investigate the long‐term impact of social distancing restrictions on the health and well‐being of populations who may experience disadvantage, including people with disability, ageing adults, and people with income insecurity. Health and social service providers need to be well resourced (financially and professionally) to address the health consequences which emerge due to social distancing restrictions. They must also be supported with the knowledge and training to provide appropriate care to priority populations experiencing distinct health and social consequences which differ from the general public.

## AUTHOR CONTRIBUTIONS

Dr Ali Lakhani is the lead in all aspects of this study and the development of the manuscript including: study conceptualization, data collection and analysis and writing for publication. Salvatore Dema, Josh Hose and Nazim Erdem co‐led the survey development, led recruitment and contributed to finding interpretations and the development of implications. Dr Peter Grimbeek and Dr Dennis Wollershiem respectfully assisted with data analysis and data collection, and both assisted with interpreting the findings. Dr Susan Charlifue provided a critical review of the manuscript and provided implications and discussion points.

## CONFLICT OF INTEREST

The authors have no conflict of interest to declare.

## Data Availability

Data underpinning this study has not been made publicly available.
